# Medical Facts and Observations upon Diseases

**Published:** 1841-07

**Authors:** 


Art. XIV.—*Medizinische Zustände und Forchungen im Reiche der Krankheiten.* Von Dr. Robert Volz.—1839. 8vo, pp. 256.
*Medical Facts and Observations upon Diseases.* By Dr. Robert Volz.

This work, as the title implies, is divided into two parts; and these quite unconnected with each other. In the first Dr. Volz, although disclaiming the character of an historical writer, indulges the reader with "a review of the past state of medicine, as the best means of judging of the present." This he represents as some horrible monster, some dragon of Wantley, "monstrum, informe, ingens," dragging its huge length through the land, whose tail is the "médecine physiologique," and "homopathie" its teeth and claws. He thinks the theory of Broussais may be considered as a sort of moral cholera, which first upraised its hydra head in France. "That country," says he, "accustomed for a quarter of a century to revolutions, wars, and fearful blood-sheddings, had fallen into such a state of congestion, that Broussais became an instrument in the hands of Nature to save his country." Dr. Volz doubts whether Napoleon or Broussais cost their country most blood! As some salvo however to the conscience of the latter, he instances "the rich results of pathological anatomy" with which he has stored our museums.

The second portion of the work before us is practical. In facial neuralgia, the author recommends the internal use of stramonium in decoction. He extols the nitrate of silver in increased cardiac action, passive hemorrhages, and irregular menstruation; and speaks highly of an ointment of the nitrate of lead as very beneficial in excoriated nipples.

